# Integrated Analysis of Bulk RNA Sequencing, eQTL, GWAS, and Single‐Cell RNA Sequencing Reveals Key Genes in Hepatocellular Carcinoma

**DOI:** 10.1111/jcmm.70359

**Published:** 2025-01-23

**Authors:** Mingkai Gong, Xian Zhao, Qingze Li, Qisheng Hao, Lichao Cha, Guofei Dong, Xinyu Li, Fabo Qiu, Dan Li, Lantian Tian

**Affiliations:** ^1^ Department of Hepatobiliary and Pancreatic Surgery The Affiliated Hospital of Qingdao University Qingdao China; ^2^ Department of Breast Surgery Second Affiliated Hospital of Shandong First Medical University Taian China; ^3^ Department of Physiology, School of Basic Medical Sciences Shandong Second Medical University Weifang China

**Keywords:** eQTL, hepatocellular carcinoma, Mendelian randomisation, single‐cell sequencing, tumour microenvironment

## Abstract

Hepatocellular carcinoma (HCC) poses a continual therapeutic challenge owing to its elevated incidence and unfavourable prognosis, underscoring the critical need for the discovery of new molecular targets for detection and therapy. This work included the analysis of three publically accessible HCC datasets from TCGA and GEO. Instrumental variables (IVs) were derived via expression quantitative trait loci (eQTL) analysis, then followed by two‐sample Mendelian randomisation (MR) analysis utilising publically available summary statistics. Key disease‐associated genes were identified by assessing odds ratios and connecting them with differentially expressed genes across the datasets. The potential molecular mechanisms of these genes were clarified by functional enrichment analysis, clinical data analysis, single‐cell RNA sequencing, GSEA, immune cell infiltration, and immune checkpoint analysis. These findings were then confirmed by Western blotting, immunohistochemistry, and quantitative PCR. By synthesising the outcomes from differential analysis of the databases, we found two genes, SERPING1 and STEAP3, that may be crucial in the beginning and development of HCC. These genes have a role in vital biological pathways and functions, such as metabolic regulation and macrophage activation. The significance of immunological‐mediated processes in HCC was further highlighted by CIBERSORT analysis, which revealed a specific pattern of immune cell infiltration and the location of immunological checkpoints in the illness. The findings elucidate the molecular mechanisms of HCC and underscore critical genes implicated in its pathogenesis. SERPING1 and STEAP3 affect tumour cells and modify the tumour microenvironment (TME), indicating that targeting these genes may offer a viable immunotherapeutic approach for HCC in clinical settings.

## 1 Introduction

Primary liver cancer was the third leading cause of cancer‐related deaths globally in 2020 and the sixth most prevalent kind of cancer overall [1]. Hepatocellular carcinoma (HCC), the predominant form of liver cancer, accounts for around 80% of all cases [2]. Despite the availability of surgical intervention for patients with early‐stage liver cancer, the majority are detected at an advanced stage, resulting in postoperative recurrence and metastasis, accompanied by a dismal prognosis [3].

Advanced‐stage liver cancer is often associated with high molecular heterogeneity, poor response to conventional therapies, and limited treatment options, further complicating disease management. Moreover, the lack of effective biomarkers for early detection and personalised treatment significantly contributes to the high mortality rate [4, 5]. Systemic treatment is very crucial for those with advanced liver carcinoma. Approximately 50% of liver cancer patients have systemic therapy, with half originally diagnosed with advanced HCC and the other half receiving treatment after HCC progression. Notwithstanding progress in HCC research, considerable obstacles remain, especially in comprehending tumour regulation, formulating early diagnostic methodologies, and executing personalised precision medicine strategies [6].

The tumour microenvironment (TME), which often has an immunosuppressive trait, is crucial to the development of HCC. Stromal elements, including macrophages and cancer‐associated fibroblasts, restrict immune cell penetration and facilitate tumour immune evasion [7], hence diminishing the efficacy of immunotherapy. The recognition of immunological checkpoints between neoplasms and immune cells has revolutionised cancer immunotherapy. Immune checkpoint drugs have revitalised anticancer immune responses by interrupting co‐inhibitory T cell signalling, transforming therapy strategies for several malignancies in the last decade [8].

Mendelian randomisation (MR) is a methodological approach used in epidemiological research to evaluate causal links. In order to evaluate the causal connections between these exposures and results, it employs genetic variants that exhibit a robust association with environmental conditions as instrumental variables [9]. By using the random distribution of alleles, MR mitigates confounding effects from unmeasured variables, such as lifestyle and environmental factors [10]. GWAS, or genome‐wide association studies, is a crucial method for discovering correlations between genomic areas and phenotypes or illnesses, providing insights into underlying molecular mechanisms. GWAS have been extensively utilised across various fields, including toxicology and food science, to uncover genetic determinants of phenotypic variability and disease susceptibility [11, 12]. By bridging the gap between genotype and clinical outcomes, their use in HCC holds the promise of discovering new biomarkers and therapeutic targets. Expression quantitative trait loci (eQTL) are chromosomal regions that clarify the relationship between a gene's expression levels and genetic variation [13]. eQTL is essential for elucidating the processes of genomic loci linked to illnesses and has effectively discovered many loci predisposed to HCC [14]. This research enhances conventional MR analysis with the use of colocalization analysis of eQTL and GWAS data to pinpoint probable critical genes linked to HCC [15]. This study used a three‐step SMR technique to analyse possible causal links between eQTL‐associated genes and HCC. We used the Heterogeneity in Dependent Instrument (HEIDI) test to determine whether the observed connections were due to pleiotropy. Furthermore, we performed several sensitivity studies to investigate how strong these associations were and to examine the interaction between genotypes and gene expression levels [16].

This work conducts a comprehensive examination of the complex processes underlying HCC, using data from several databases and combining multi‐omics methodologies to identify new treatment targets. The primary goals were to analyse microarray datasets in order to determine which genes (DEGs) are expressed differently in HCC than in normal tissues. Additionally, we used eQTL and MR investigations to evaluate the association and causal relationships between these genes and the pathophysiology of HCC. We observed the silencing of SERPING1 and STEAP3 in hepatocellular carcinoma tissues—genes that significantly impact clinical outcome. The correlation between SERPING1 and STEAP3 expression and numerous clinicopathological characteristics was further investigated. Additionally, possible signalling pathways and biological activities associated with these genes were found using gene set enrichment analysis (GSEA), Gene Ontology (GO), and the Kyoto Encyclopedia of Genes and Genomes (KEGG). We evaluated their interaction with the tumour immune milieu, providing insights that may inform future therapeutic therapy. These results indicated that SERPING1 and STEAP3 had a salient predictive value and could be candidate targets for immunotherapeutic intervention against HCC.

## 2 Methods and Materials

### 2.1 Data Gathering

From the TCGA dataset, clinicopathological and RNA‐seq expression data were acquired for 50 nearby nontumor tissue samples and 374 HCC samples. Age, gender, clinical stage, T stage, N stage, M stage, and follow‐up status are among the clinical features of this group's transcriptome profile. Probe annotations were matched to corresponding Entrez gene ID by using reference annotation files. The GSE14520 dataset from the GEO was specifically selected for this study based on the following criteria: (1) relevance to HCC, including tumour and adjacent non‐tumour tissues; (2) availability of detailed clinical annotations, such as survival data and tumour staging, to facilitate clinical correlation analyses; (3) a sufficiently large sample size (488 samples across two platforms), providing robust statistical power; and (4) compatibility of the platforms (GPL571 and GPL3921) with accurate probe annotation for transcriptomic analysis. Additionally, this dataset has been widely validated and utilised in prior HCC research, further supporting its selection for integrative analyses. In Supplementary Table 1, the clinical characteristics of participants from the TCGA and GSE14520 cohorts are compiled.

The GWAS collection (https://www.eqtlgen.org/phase1.html) provided the eQTL data utilised in this study, which included 19,942 genes. Single nucleotide polymorphisms (SNPs) strongly correlated with gene expression (*p* < 5e−08) were designated as instrumental variables using the R package “TwoSampleMR.” Linkage disequilibrium was regulated using parameters established at *r* ^2^ < 0.001 and a clustering distance of 10,000 kb.

**FIGURE 1:**
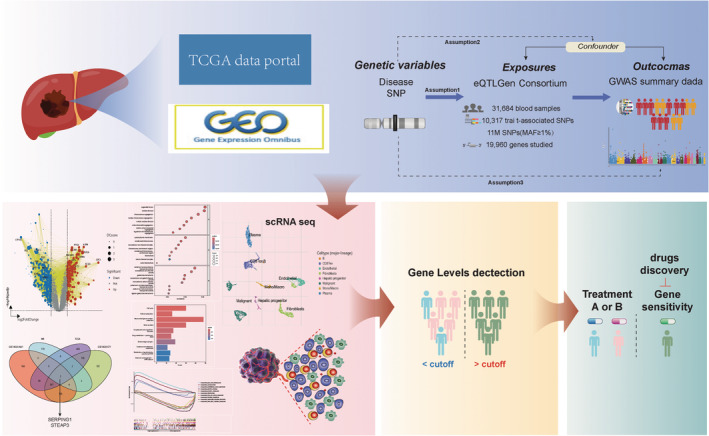
This flowchart illustrates the significance of SERPING1 and STEAP3 to HCC.

To exclude SNPs with poor correlations or inadequate explanatory power for phenotypic variation, a “*F*‐test threshold of > 10” was used. Jiang et al. reported 456,348 cases in the largest GWAS on HCC, from which outcome data were gathered [14]. The GWAS catalogue (https://www.ebi.ac.uk/gwas/) provided access to the data. In Figure 1, the whole study process is shown.

### 2.2 Screening of Instrumental Variables (IVs) and MR Analysis

SNPs that were substantially associated with HCC were identified using a genome‐wide significance threshold of *p* < 5 × 10^−8^. Linkage disequilibrium (LD) was reduced by establishing the LD parameter (*r* ^2^) at 0.001 and a genetic distance criterion of 10,000 kb, so assuring the independence of the chosen SNPs. The F statistic was used to evaluate the link strength between instrumental variables and exposure factors, retaining those SNPs with *F* values over 10 to mitigate bias from weak instruments [17, 18]. To investigate the connections between candidate genes and HCC, MR research was conducted using the “TwoSampleMR” program and the inverse variance weighting (IVW) method. The weighted median, weighted mode, simple mode, and MR‐Egger methods were used for further sensitivity analyses. We used three criteria to identify genes associated with disease: First, genes in the IVW analysis with *p* < 0.05 were selected; second, genes were validated based on consistent directional effects (Odds Ratio values) across three MR techniques; and third, genes with *p* < 0.05 that showed signs of pleiotropy were excluded.

To address the potential bias introduced by horizontal pleiotropy, we applied MR‐Egger regression, which includes an intercept test to evaluate directional pleiotropy. Non‐significant intercept values (*p* > 0.05) suggest that horizontal pleiotropy is unlikely to influence the results. For further sensitivity analyses, weighted median, weighted mode, and simple mode techniques were used. These methods guarantee the validity of the causal estimations and are robust to pleiotropic effects. To mitigate the risk of sample overlap, we ensured that exposure data and outcome data were sourced from independent cohorts. While sample overlap cannot be entirely excluded, the *F*‐statistics for instrumental variables surpassed 10, reducing the likelihood of weak instrument bias. These measures ensure that the results are robust and minimally affected by horizontal pleiotropy or sample overlap.

### 2.3 DEGs Identification

Version 4.2.2 of the R program was used to read and prepare data related to liver cancer from two platforms, GPL571 and GPL3921, in the GSE14520 dataset. Differential expression analysis was performed independently for normal and liver cancer tissues. The significance criterion for differentially expressed genes (DEGs) was established at *p*‐adjust ≤ 0.05 and |log2 Fold Change| ≥ 1. The “pheatmap” program was used to generate volcano plots and heat maps of the DEGs [19]. Cross‐referencing was used to identify genes that were co‐expressed in the MR analysis and DEGs. To determine their possible causal relationships with HCC, MR analysis was then performed on all of the overlapped genes. For robustness and dependability, sensitivity analyses excluding one, pleiotropy tests, and heterogeneity tests were performed. Funnel plots, forest plots, and scatter plots were generated to support the visualisation of these results. With the TIMER database, we explored the expression of DEGs across cancer types and obtained a comprehensive overview.

### 2.4 Analysis of GO/KEGG Enrichment

The BioConductor tool “clusterProfiler” was used to analyse the GO and KEGG pathways on the intersecting genes. The significance levels were set to be very stringent: less than a false discovery rate of 0.05 and less than a *p*‐value of 0.05.

### 2.5 Analysis of Gene Set Enrichment (GSEA)

After the samples were separated into groups with high and low expression based on the median expression levels of the relevant genes, differential expression analysis was conducted using the “limma” R program. All genes were ranked in order of importance based on logFoldChange values gathered from this investigation. GSEA was conducted using 1000 permutations through GSEA function implemented in the “clusterProfiler” R package. Analyses were focused on the HALLMARK gene collection. The resultant data were further visualised via functions “ridgeplot” and “gseaplot2” toward insightful and interpretable results.

### 2.6 Single‐Cell Sequencing Data Analysis

Hepatocellular carcinoma scRNA‐seq data are retrieved from a study entitled TISCH, a platform for single‐cell transcriptomics in oncology. Single‐cell sequencing with high throughput has been performed, providing an overall analysis of the cell composition and transcriptomic landscape of HCC, thus indicating heterogeneity in the tumour microenvironment. We used scRNA‐seq data in the HCC tissue, GSE125449, and then compared our data with the TISCH database, further exploring some genes for their expression levels in specific cell types within HCC tissues.

### 2.7 Co‐Expressed Genes Analysis in HCC

To get a more profound understanding of the biological relevance of intersectional genes in HCC, the genes underwent further analysis using the Pearson correlation test, with filtering criteria established at absolute corFilter > 0.4 and *p*_Filter_ = 0.001.

### 2.8 Immune Cell Analysis

22 immune cell types had their enrichment scores calculated using the CIBERSORT technique. The expression levels of SERPING1 and STEAP3 were evaluated in relation to immunological checkpoints such as CTLA4, PDCD1/PD‐1, and CD274/PD‐L1, as well as the degree of immune cell infiltration, employing the study of Spearman correlation. Using the Wilcoxon rank‐sum test, the levels of immune cell infiltration between the high‐expression and low‐expression groups were assessed.

### 2.9 Drug Sensitivity Tests Analysis

To predict anticancer drugs' half maximal inhibitory concentration (IC50), the R package “pRRophetic” was used [20], indicating the effectiveness of a compound in obstructing a biological process or organism. Both groups of important genes with high and low expression were predicted.

### 2.10 Cell Culture, Reagents, and Quantitative Polymerase Chain Reaction (qPCR)

The hepatocyte cell line WRL68 and the HCC cell lines HepG2, Hep3B, HuH‐7, HCCLM3, and SNU‐387 were obtained from Qingdao University's Affiliated Hospital. All cell lines were authenticated by short tandem repeat (STR) profiling and confirmed to be free of mycoplasma contamination [21]. Total RNA was extracted using the TRIzol (Invitrogen, USA), and the quantity was measured using a NanoDrop. RNA samples having A260/A280 ratios between 1.8 and 2.0 were reverse‐transcribed into cDNA using the PrimeScript kit (Takara, Japan). A CFX96 Real‐Time PCR System (Bio‐Rad, USA) was used to perform quantitative PCR (qPCR) using the TB Green kit (Takara, Japan). The 2^−ΔΔCt^ technique was used to measure the data, while GAPDH served as the internal reference. Table 1 contains all of the primer sequences used for RT‐qPCR in this experiment.

**TABLE 1:** Sequences of gene‐specific primers used for real‐time RT‐qPCR.

Gene	Forward primer (5′–3′)	Reverse primer (5′–3′)
GAPDH	GAGAAGGCTGGG GCTCATTT	TGATGACCCTTT TGGCTCCC
SERPING1	CGTGGCCCGAAACTTACTCA	TGGCAGTGCTTACTCAAGCC

### 2.11 Western Blot (WB)

The cells were first lysed in the culture plate, subsequently collected, and lysed on ice. The supernatant was collected and analysed using gel electrophoresis. Following the transfer of proteins to membranes, they were incubated with particular primary and secondary antibodies, then washed to eliminate non‐specifically bound antibodies before to detecting development.

### 2.12 Immunohistochemistry (IHC)

Clinical specimens were obtained, encased in paraffin, then deparaffinised using xylene and graded ethanol. Thereafter, the slices were treated with H_2_O_2_ to deactivate endogenous peroxidase activity. The samples were then submerged in an antigen retrieval solution and subjected to boiling for 20 min. Following blocking with goat serum, the sections were treated with biotinylated goat anti‐rabbit IgG polymer after being incubated with SERPING1 antibody for a whole night. The slices were subsequently developed with DAB, washed with distilled water, counterstained with haematoxylin, and then differentiated using a differentiation solution.

### 2.13 Lentivirus Packaging

Plasmids for lentiviral packaging were acquired from TongYong, located in Shanghai, China. HCC cells were cultivated for 24 h after being seeded at a density of 2 × 10^5^ cells per plate prior to transduction. Recombinant lentiviruses carrying either SERPING1 overexpression constructs or SERPING1 knockdown sequences (shSERPING1) were used to infect the cells. For 48 h following infection, the cells were maintained in complete media supplemented with 2 μg/mL puromycin in order to select for populations that were successfully transduced. The cells were subsequently harvested for Western blot analysis to confirm transgene expression.

### 2.14 Transwell and Wound‐Healing Assays

Transwell chambers (Corning, USA) were used for the tests of cell invasion and migration. Matrigel (Corning, USA) was frozen overnight at 4°C for the invasion experiment. To allow for solidification, a 60 μL Matrigel mixture diluted in DMEM was added to the top chamber and incubated for 20 min at 37°C. Next, 4 × 10^4^ cells suspended in 300 μL of DMEM were seeded into the top compartment, while 500 μL of media supplemented with 20% foetal bovine serum was introduced to the bottom chamber. Following a 12‐h incubation period at 37°C, the cells were fixed with 10% formaldehyde and stained for 20 min with 0.1% crystal violet. The pigmented cells on the membrane's bottom surface were counted using a microscope, while non‐invading cells were removed from the membrane's top surface using a cotton swab.

With the exception of not using Matrigel in the top chamber, the migration test was conducted in the same manner as the invasion assay. Wound healing tests were conducted using HCC cells that were seeded into 6‐well plates and allowed to reach around 90% confluency. Following the introduction of a scratch using a pipette tip, the cells were cultured at 37°C in a humidified environment with 5% CO₂ in media containing 1% foetal bovine serum. To assess the capacity for cell migration, wound closure was monitored and documented at 0, 24, and 48 h.

### 2.15 EdU Assay

According to the guidelines provided by the manufacturer, EdU staining was carried out using an EdU assay kit (Beyotime, China). In short, cells were fixed and permeabilized, then treated with 10 μM EdU for 2 h. After that, the cells were incubated for 30 min at room temperature in the dark with a click reaction solution that included copper sulphate and Azide 488. Hoechst 33342 was used to counterstain the nuclei for 10 min. A confocal microscope (Zeiss, Germany) was used to take pictures, and the proportion of EdU‐positive cells was calculated to assess rates of proliferation.

### 2.16 Immunofluorescence (IF)

Following cell adhesion to coverslips, the cells were fixed and permeabilized for 10 min. PBS was then used for washing. Following blocking, cells were treated at 4°C for a whole night with primary antibodies. The cells were incubated on a shaker in the dark for an hour the next day after secondary antibodies that had been fluorescently labelled were added. The nuclei were counterstained with 4′,6‐diamidino‐2‐phenylindole (DAPI). Images of immunofluorescence were taken using a Zeiss fluorescence microscope (Zeiss, Germany).

### 2.17 Statistical Analysis

R software (version 4.2.2) and GraphPad Prism 9 were used for statistical analysis. The survival distributions of each group were compared using the log‐rank test and Kaplan–Meier calculation for survival analysis. The unpaired *t*‐test was used to evaluate regularly distributed data in pairwise comparisons, while the Mann–Whitney *U*‐test was used to investigate non‐normally distributed data. The Kruskal‐Wallis test was used when comparing more than two groups. Spearman's rank correlation coefficient was used for correlation analysis.

G*Power (version 3.1) was used for post hoc power analysis for important statistical tests in order to guarantee the validity of our results. Our study's statistical power (> 0.8) to identify significant differences was validated by the analysis. Results showed that observed differences were both statistically and physiologically significant, according to effect sizes computed for the Kruskal‐Wallis tests (eta‐squared) and *t*‐tests (Cohen's *d*). Furthermore, sensitivity studies were performed to investigate the durability of our findings. To determine if the observed relationships were consistent, robustness tests were conducted on the data and potential confounding factors were evaluated.

When the p‐value for any two‐tailed test was less than 0.05, it was considered statistically significant. R programs were used to visualise the data and display the statistics (ggplot2).

## 3 Result

### 3.1 Overview of the Datasets

**FIGURE 2:**
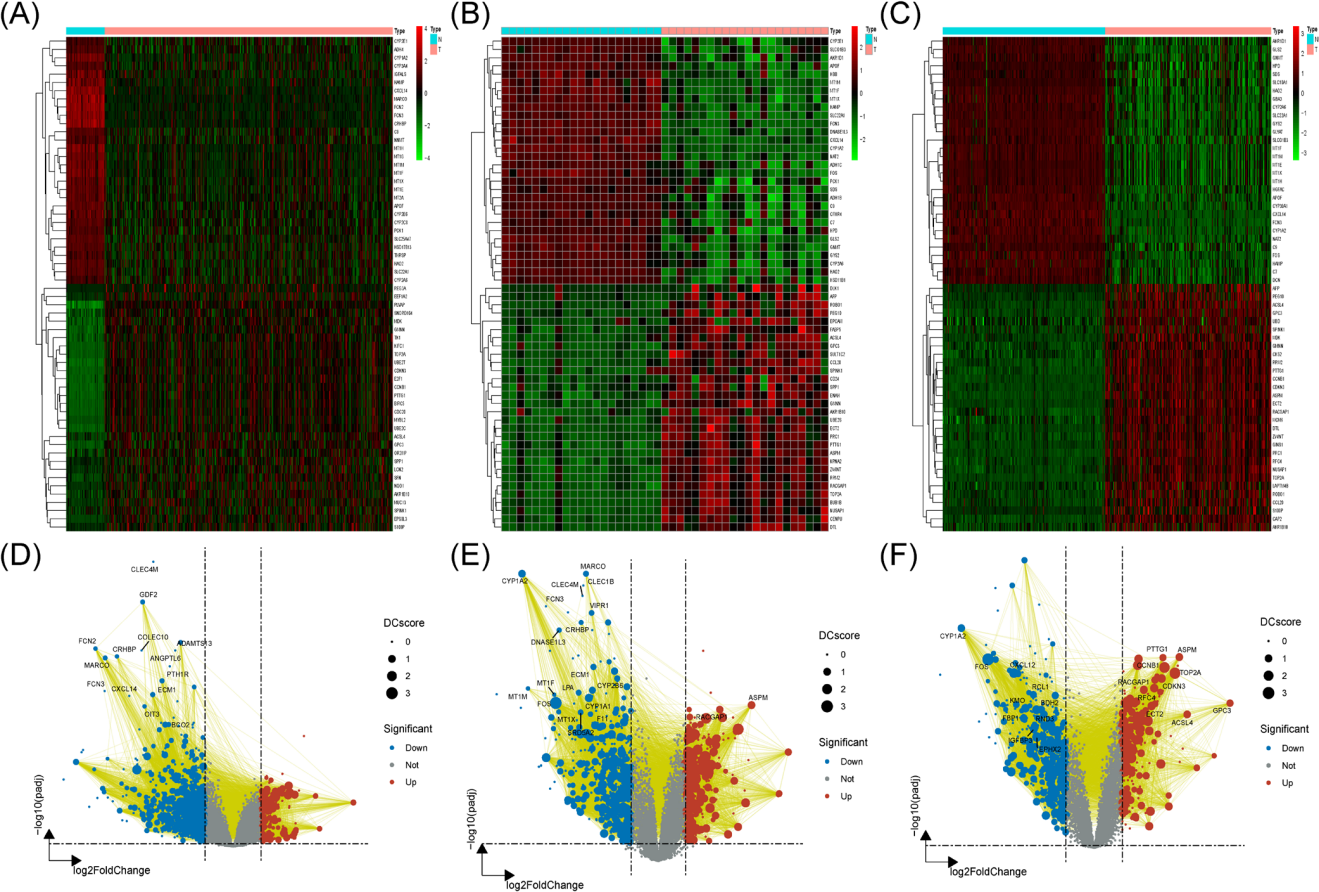
The heat maps and volcano plots of datasets. (A) Heat map of LIHC in TCGA. (B) Heat map of GSE14520 in GPL571. (C) Heat map of GSE14520 in GPL3921. (D) TCGA volcano plot. (E) GSE14520 in GPL571 volcano plot. (F) GSE14520 in GPL3921 volcano plot.

The GSE14520 dataset's differential expression analysis of the GPL3921 platform discovered 779 DEGs, while the GPL571 platform analysis uncovered 854 DEGs. Furthermore, data from 374 hepatocellular carcinoma cases and 50 normal samples were obtained from TCGA. Heat maps and volcano plots were generated using fold‐change values and modified *p*‐values. In the heat maps, red signifies elevated DEGs, while green denotes downregulated DEGs. Due to the substantial quantity of DEGs, the 30 most upregulated and 30 most downregulated genes were presented. In the volcano plots, red points signify highly elevated DEGs, while blue points indicate considerably downregulated DEGs. Heat maps (Figure 2A–C) and volcano plots (Figure 2D,E) for these three datasets are shown. Following MR screening, 26,152 SNPs were recognised as instrumental variables, all satisfying the three essential criteria of MR, with *F*‐statistics surpassing 10 for each chosen SNP (as shown in Supplementary Table 2). According to MR analysis and the three established screening criteria, 190 genes associated with HCC were found (detailed in Supplementary Table 3).

### 3.2 DEGs Identification

**FIGURE 3:**
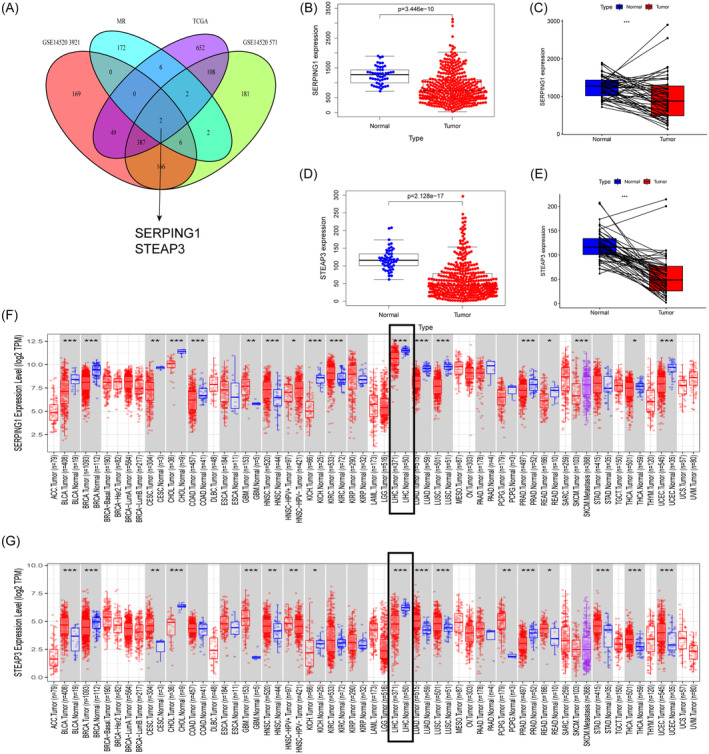
SERPING1 and STEAP3 expression pattern in normal and tumour tissues. (A) The Venn diagram illustrates the overlapping genes shared among the four cohorts. (B) SERPING1 mRNA levels in HCC samples and normal liver samples. (C) The expression level of SERPING1 in HCC samples was markedly lower compared to that in the corresponding non‐cancerous adjacent tissues. (D) STEAP3 mRNA levels in HCC samples compared to those in normal liver tissues. (E) The expression of STEAP3 in HCC samples was significantly reduced compared to that in the corresponding non‐cancerous adjacent tissues. (F–G) The level of SERPING1 and STEAP3 expression in different tumour types analysed in TIMER2.0. *p < 0.05, **p < 0.01, ***p < 0.001. ns, no significance.

Utilising crossover analysis, we identified two crossover genes between disease‐related genes and DEGs, namely SERPING1 and STEAP3, as seen in Figure 3A. The two genes' expression levels in normal tissues and HCC were compared using TCGA. Figure 3B,C illustrates the tissue expression levels and paired differential analysis of SERPING1, indicating a tendency of low expression. Figure 3D,E illustrates the tissue expression levels and paired differential analysis of STEAP3, indicating a tendency of low expression. The TIMER online analysis database was used to examine the expression of SERPING1 and STEAP3 in different types of cancer. The findings indicated that SERPING1 and STEAP3 were aberrantly regulated in the majority of tumours. SERPING1 exhibited downregulation in the majority of cancer types, such as colorectal cancer (COAD), lung squamous cell carcinoma (LUSC), lung adenocarcinoma (LUAD), and HCC, whereas it was increased in select malignancies, notably renal clear cell carcinoma (KIRC) (Figure 3F). Most cancers, including head and neck squamous cell carcinoma (HNSC) and bladder cancer (BLCA), have increased STEAP3, whereas it was downregulated in some cancers, such as breast cancer (BRCA) (Figure 3G).

### 3.3 MR Analysis

**FIGURE 4:**
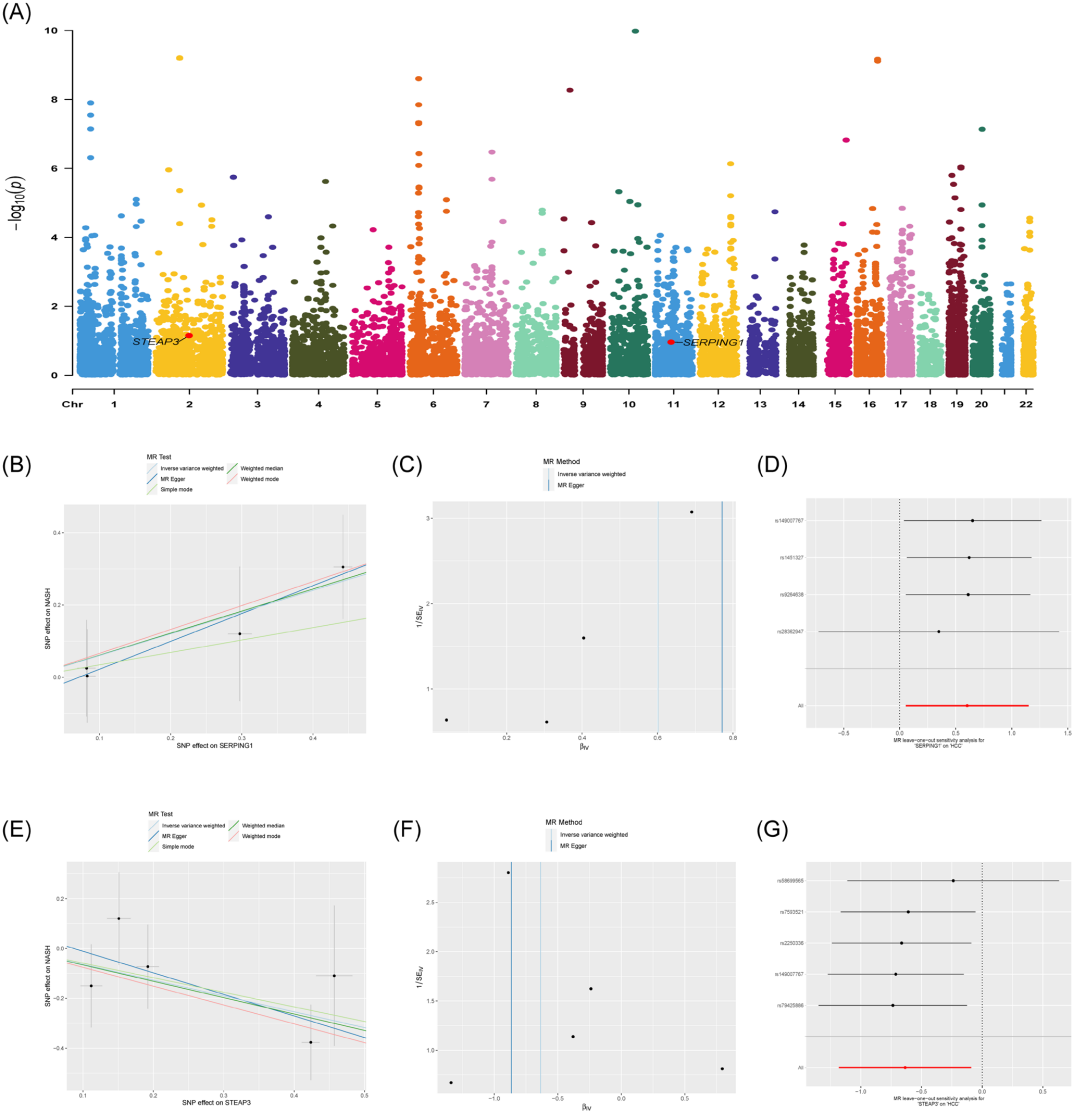
Results from the Mendelian randomisation study examining the relationship between SERPING1, STEAP3, and HCC. (A) Manhattan plot of MR analysis in discovery phase. SERPING1 and STEAP3 were labelled and highlighted in red. (B, E) Scatter plots illustrating the effects of individual SNPs and estimates derived from various Mendelian randomisation techniques for SERPING1 and STEAP3 in relation to HCC. (C, F) Funnel plots of SERPING1 and STEAP3 on HCC. (D, G) Leave‐one‐out analysis plots for SERPING1 and STEAP3 on HCC. MR, Mendelian randomisation; SNP, single‐nucleotide polymorphism.

To further our comprehension of the chromosomal distribution of these genes, we depicted their relative positions, as seen in Figure 4A. MR analysis was performed on SERPING1 and STEAP3 to evaluate their causal association with HCC. While STEAP3 showed a negative causal link with HCC (OR = 0.530; 95% CI: [0.307 to 0.915]; *p* = 0.02), there was a significant positive causal connection between SERPING1 and HCC (OR = 1.83; 95% CI: [1.055 to 3.158]; *p* = 0.03) according to the IVW approach in MR analysis (Table 2). For further validation, MR‐Egger, weighted median, weighted mode, and simple mode approaches were used; the weighted median also suggested that these genes lower the incidence of HCC (Figure 4B,E). Tests for heterogeneity and pleiotropy yielded non‐significant findings (*p* > 0.05), indicating that the effects of heterogeneity and pleiotropy may be disregarded (Figure 4C,F). The robustness of the findings was validated by the sensitivity analysis of leave‐one‐out, since the impact sizes of distinct instrumental factors were highly correlated with the total effect size (Figure 4D,G).

**TABLE 2:** MR analysis of co‐expressed genes.

Exposure	nsnp	Method	*p*	OR (95% CI)
SERPING1	4	Weighted median	0.036	1.842 (1.040–3.262)
	4	Inverse variance weighted	0.032	1.825 (1.055–3.158)
STEAP3	5	Weighted median	0.041	0.518 (0.276–0.974)
	5	Inverse variance weighted	0.023	0.530 (0.307–0.915)

### 3.4 SERPING1 and STEAP3 Clinicopathological Traits and Prognosis in HCC: Insights From Analysis of TCGA Database

**FIGURE 5:**
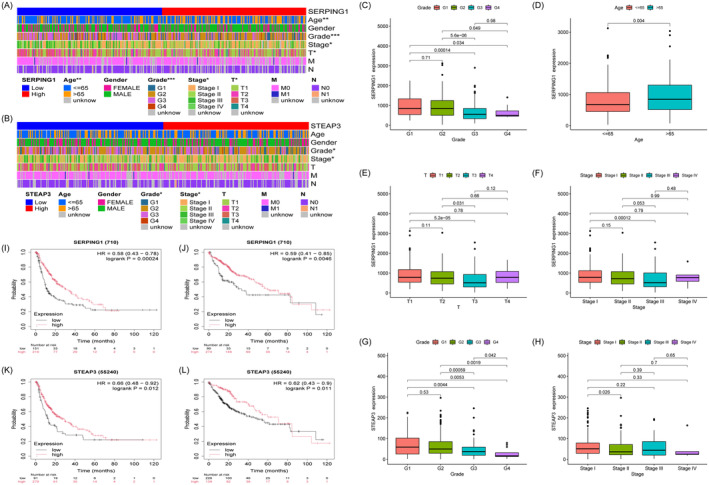
Correlation between gene expression and HCC clinicopathological data as well as prognosis. (A, B) Expression‐clinical parameters heatmap of SERPING1 and STEAP3 in TCGA datasets. (C–F) SERPING1 expression in HCC tissues with different grade, age, T‐classification and Stages. (G, H) STEAP3 expression in HCC tissues with different grade and T stages. (I, J) Kaplan–Meier curve showing differences of OS and PFS between low‐ and high‐SERPING1 expression HCC patients. (K, L) Kaplan–Meier curve showing differences of OS and PFS between low‐ and high‐STEAP3 expression HCC patients. *p < 0.05, **p < 0.01, ***p < 0.001; OS, overall survival; PFS, progression‐free survival.

To further explore the relationship between gene expression and HCC, we assessed the relationships between gene expression and clinical variables, including age, gender, grade, stage, and TNM classification. Significant differences were seen between the cohorts with high and low SERPING1 expression in terms of age, grade, stage, and T‐classification (Figure 5A), along with variations in grade and stage between the high‐ and low‐STEAP3 expression cohorts (Figure 5B). Elevated SERPING1 expression was significantly correlated with grade (G1 vs. G3, G1 vs. G4, G2 vs. G3, G2 vs. G4, *p* < 0.05, Figure 5C), age (*p* < 0.05, Figure 5D), T‐classification (T1 vs. T3, T2 vs. T3, *p* < 0.05, Figure 5E), and clinical stage (Stage I vs. Stage III, *p* < 0.05, Figure 5F). Increased STEAP3 expression exhibited a significant correlation with grade (G1 vs. G3, G1 vs. G4, G2 vs. G3, G2 vs. G4, G3 vs. G4, *p* < 0.05, Figure 5G) and clinical stage (Stage I vs. II, *p* < 0.05, Figure 5H). According to the findings, clinical progression in patients with HCC is correlated with SERPING1 and STEAP3 expression levels. Kaplan–Meier (K‐M) curves indicated that individuals exhibiting elevated SERPING1 and STEAP3 expression had enhanced overall survival (OS) (Figure 5I,K). Progression‐free survival (PFS), an essential measure of quality of life in malignant tumours, was elevated in the high‐expression cohorts of SERPING1 and STEAP3 significantly compared to low‐expression cohorts (Figure 5J,L), indicating that SERPING1 and STEAP3 may function as protective prognostic markers in HCC progression.

### 3.5 The Expression of SERPING1 and STEAP3 in Cells With scRNA‐Seq Analysis

**FIGURE 6:**
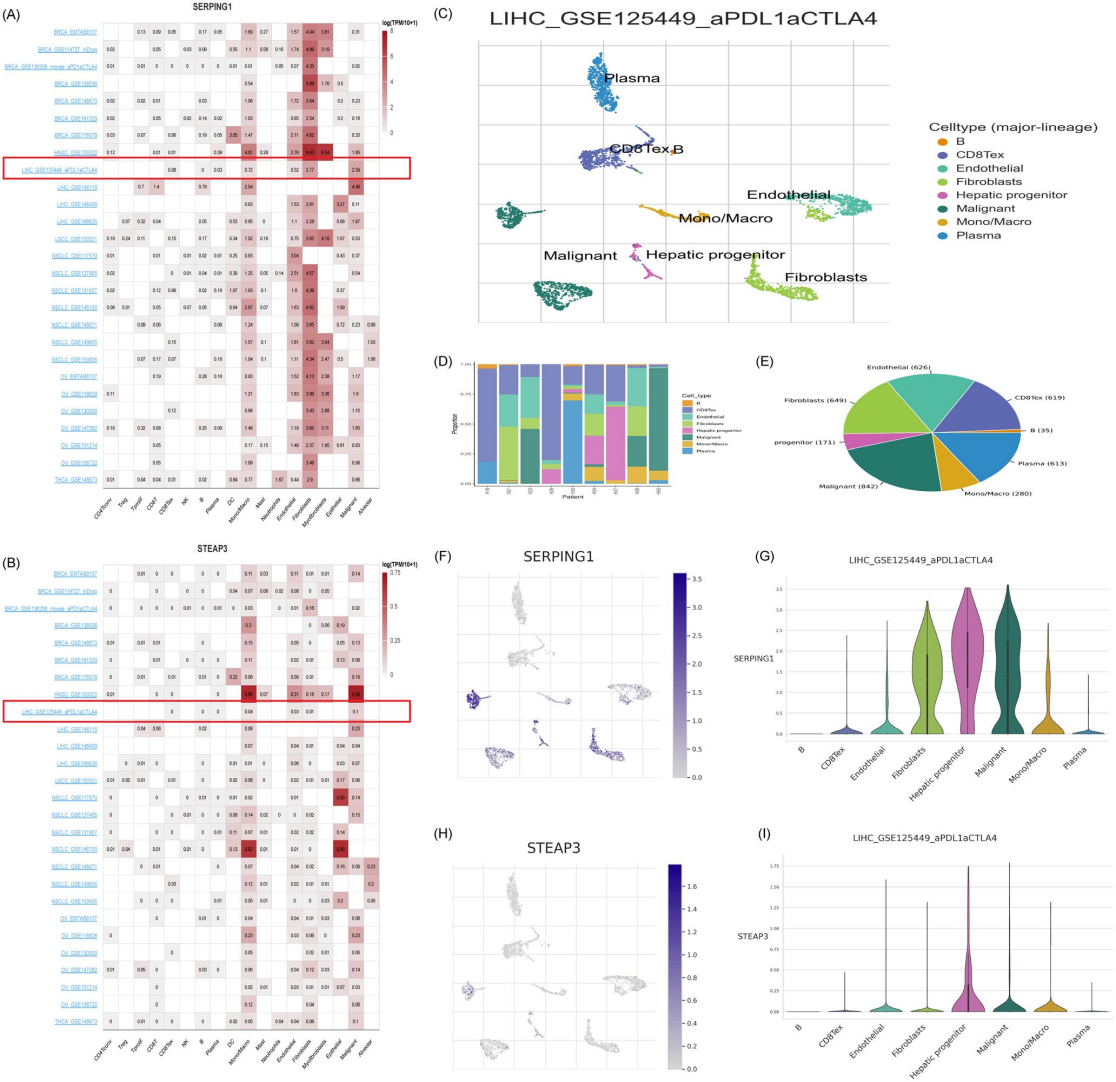
Single‐cell analysis of SERPING1 and STEAP3 in cancers. (A, B) Levels of SERPING1 and STEAP3 expression of 18 cell types in 27 single‐cell datasets were assessed and visualised in a heatmap. (C) Uniform Manifold Approximation and Projection (UMAP) plots of all single cells of HCC patients, showing all cell types in the plot. (D, E) Displays the relative proportions of each cell type identified in the public dataset, as well as the proportion of integrated immune cells included in the database. (F, G) T‐SNE projection illustrating the distribution of all cells alongside the expression levels of SERPING1 derived from the dataset. (H, I) T‐SNE projection illustrating the distribution of all cells alongside the expression levels of STEAP3 derived from the dataset.

We conducted single‐cell analysis using cancer sample files from the TISCH database to identify the principal cell types that express SERPING1 and STEAP3 inside the TME. A heatmap illustrating SERPING1 and STEAP3 expression across 18 cell types demonstrated that SERPING1 was mostly expressed in fibroblasts, monocytes/macrophages, endothelial cells, and myofibroblasts (Figure 6A). Conversely, STEAP3 expression was mostly seen in monocytes/macrophages, epithelial cells, and neoplastic cells (Figure 6B). To assess the transcriptional patterns of SERPING1 and STEAP3 in HCC at the single‐cell level and examine cellular heterogeneity within the HCC microenvironment, we analysed the publically accessible scRNA‐seq dataset GSE125449. This study included nine samples from GSE125449, visualised using UMAP plots, which identified and illustrated eight unique cell types (Figure 6C–E). Following quality control and batch effect correction, a total of 3834 cells were examined. Cell type‐specific indicators were identified for each cluster based on the highest‐ranked differentially expressed genes. Our findings indicate that SERPING1 is mostly expressed in fibroblasts, hepatic progenitor cells, and malignant cells (Figure 6F,G), while STEAP3 is predominately located in hepatic progenitor cells (Figure 6H,I).

### 3.6 Functional Enrichment Analyses

**FIGURE 7:**
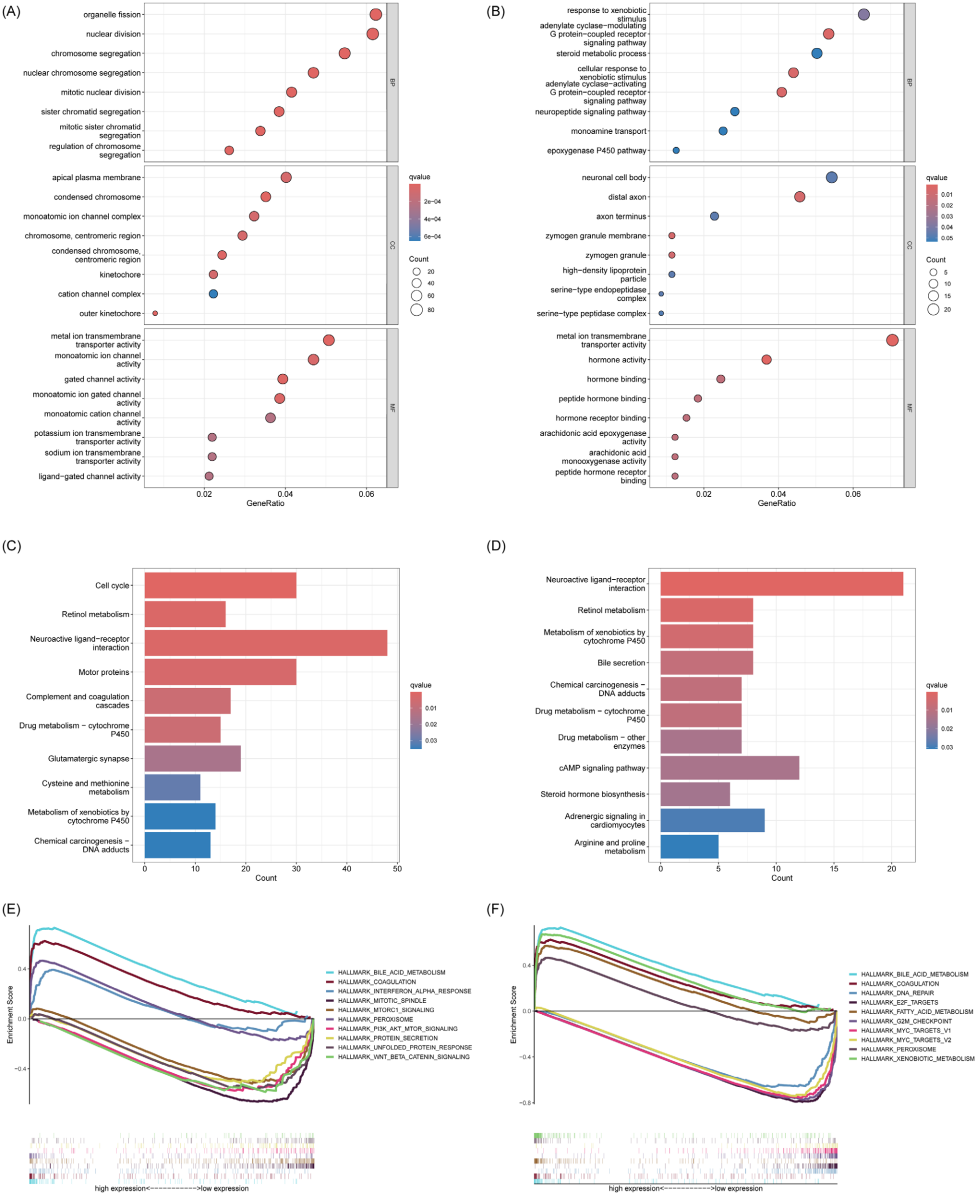
The functional analysis of SERPING1 and STEAP3 in HCC. (A, B) GO analysis of SERPING1 and STEAP3. (C, D) KEGG analysis of SERPING1 and STEAP3. (E, F) Hallmarks of SERPING1 and STEAP3. GO, gene ontology; KEGG, Kyoto encyclopedia of genes and genomes.

Utilising GO and KEGG functional enrichment analysis, the likely functions of intersected genes were examined. Within the biological process (BP) category, SERPING1 was predominantly enriched in pathways linked to nuclear division and organelle fission. In the cellular component (CC) category, it was enriched at the apical plasma membrane, whereas in the molecular function (MF) category, it was linked to metal ion transmembrane transporter activity (Figure 7A); KEGG pathway analysis indicated predominant enrichment in the cell cycle, retinal metabolism, and neuroactive ligand‐receptor interaction pathways (Figure 7C). Key keywords were linked to bile acid metabolism, coagulation, and mitotic spindle Wnt beta‐catenin signalling (Figure 7E). STEAP3 was predominantly enriched via the adenylate cyclase‐modulating G protein‐coupled receptor signalling pathway in biological process enrichment analysis, through the distal axon in cellular component enrichment analysis, and through hormone activity in molecular function enrichment analysis (Figure 7B); KEGG pathway analysis indicated that it was primarily enriched in neuroactive ligand‐receptor interaction, retinol metabolism, and the metabolism of xenobiotics by cytochrome P450 (Figure 7D). Key keywords were linked to bile acid metabolism, xenobiotic metabolism, and E2F targets (Figure 7F).

### 3.7 SERPING1 and STEAP3 Co‐Expression Networks in HCC

**FIGURE 8:**
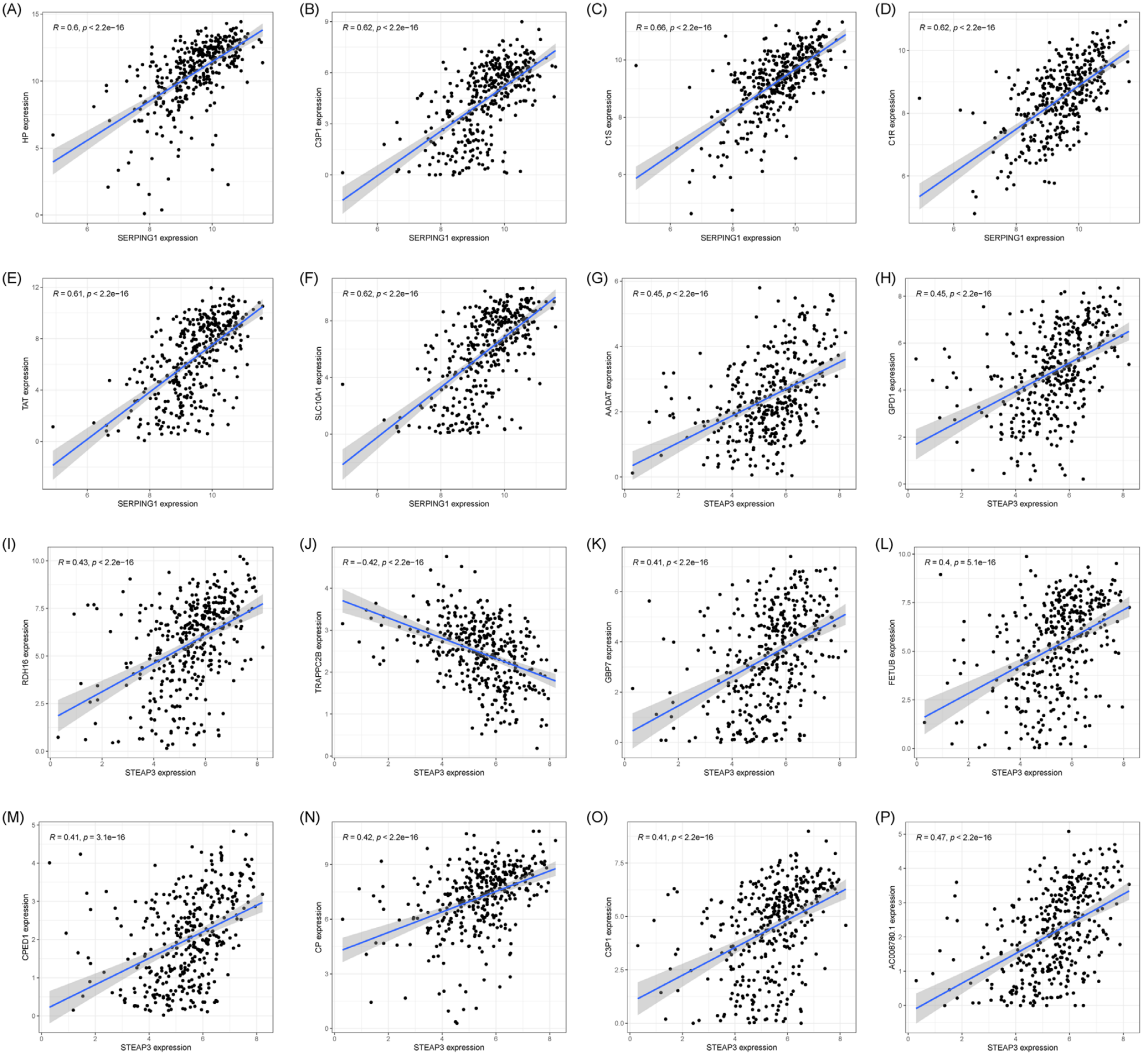
Co‐expressed genes of SERPING1 and STEAP3 in HCC. (A–F) SERPING1 was significantly correlated with HP (*r* = 0.6, *p* = 3.85e−38), C3P1 (*r* = 0.62, *p* = 7.87e−41), C1S (*r* = 0.66, *p* = 9.19e−48), C1R (*r* = 0.62, *p* = 1.77e−41), TAT (*r* = 0.61, *p* = 6.12e−40), SLC10A1 (*r* = 0.62, *p* = 1.65e−40). (G–P) STEAP3 was significantly correlated with AADAT (*r* = 0.45, *p* = 2.29e−20), GPD1 (*r* = 0.45, *p* = 1.73e−20), RDH16 (*r* = 0.43, *p* = 1.40e−18), TRAPPC2B (*r* = 0.42, *p* = 1.76e−17), GBP7 (*r* = 0.41, *p* = 1.01e−16), FETUB (*r* = 0.40, *p* = 5.06e−16), CPED1 (*r* = 0.41, *p* = 3.07e−16), CP (*r* = 0.42, *p* = 4.89e−17), C3P1 (*r* = 0.41, *p* = 5.56e−17), AC008780.1 (*r* = 0.47, *p* = 4.63e−22).

The Pearson correlation test revealed the six genes with the most significant connections with SERPING1 and the 12 genes with the most significant correlations with STEAP3. The findings indicate that SERPING1 exhibited a substantial correlation with HP (*r* = 0.6, *p* = 3.85e−38), C3P1 (*r* = 0.62, *p* = 7.87e−41), C1S (*r* = 0.66, *p* = 9.19e−48), C1R (*r* = 0.62, *p* = 1.77e−41), TAT (*r* = 0.61, *p* = 6.12e−40), and SLC10A1 (*r* = 0.62, *p* = 1.65e−40) (Figure 8A–F). STEAP3 exhibited significant correlations with AADAT (*r* = 0.45, *p* = 2.29e−20), GPD1 (*r* = 0.45, *p* = 1.73e−20), RDH16 (*r* = 0.43, *p* = 1.40e−18), TRAPPC2B (*r* = 0.42, *p* = 1.76e−17), GBP7 (*r* = 0.41, *p* = 1.01e−16), FETUB (*r* = 0.40, *p* = 5.06e−16), CPED1 (*r* = 0.41, *p* = 3.07e−16), CP (*r* = 0.42, *p* = 4.89e−17), C3P1 (*r* = 0.41, *p* = 5.56e−17), and AC008780.1 (*r* = 0.47, *p* = 4.63e−22) (Figure 8G–P). The majority of co‐expressed genes are strongly associated with the survival of HCC patients (Supporting Information FigureS1).

### 3.8 SERPING1 and STEAP3 Was Associated With Infiltrating Immune Cells in HCC Microenvironment

**FIGURE 9:**
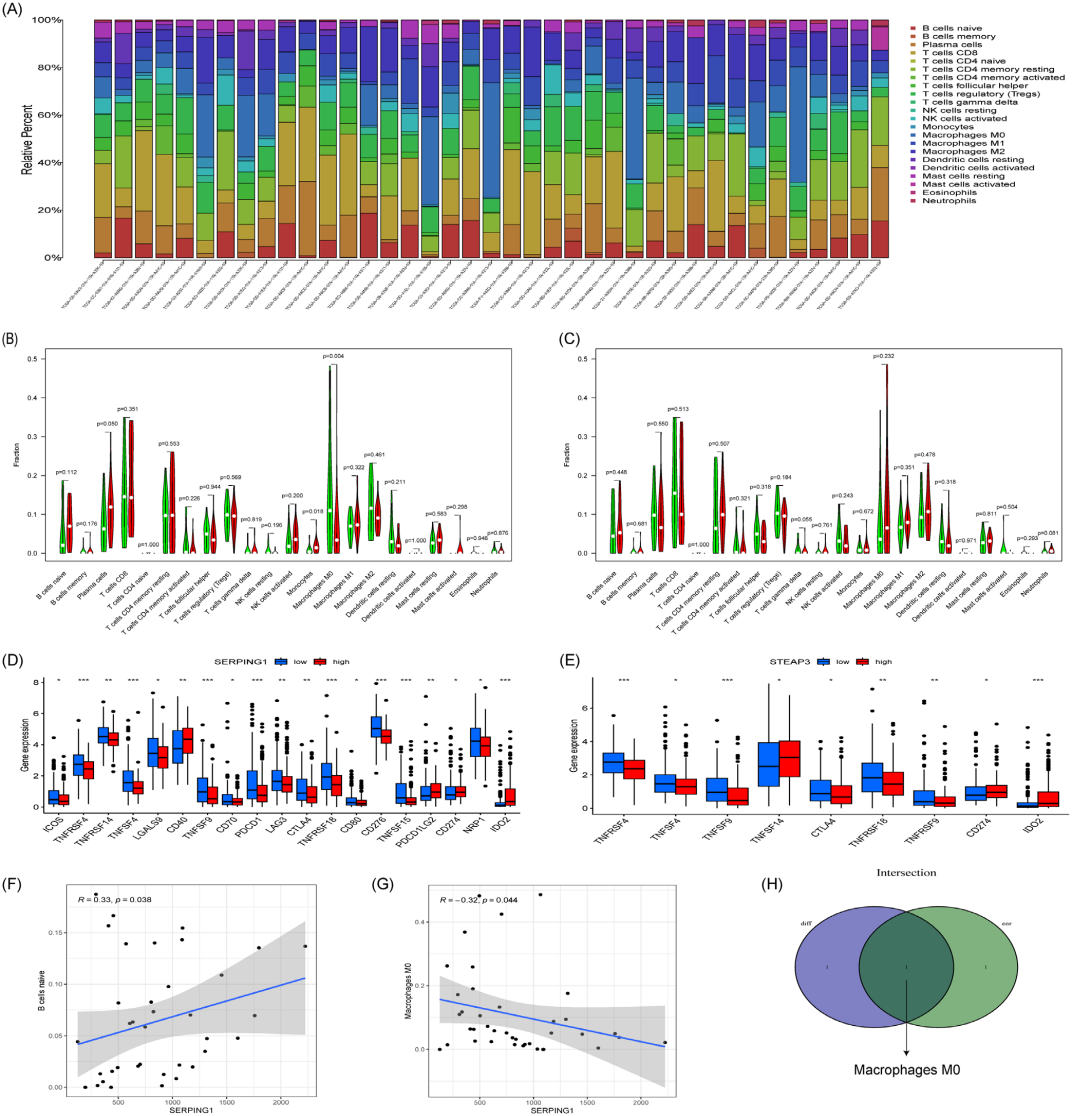
Assessment of immune cell infiltration and checkpoint in HCC. (A) Composition and relative abundance of 22 immune cell types in OA and HC samples. (B, C) Violin plot illustrating the differential analysis of immune cell infiltration associated with SERPING1 and STEAP3. (D, E) Differences in the expression of immune checkpoint genes between the high‐ and low‐expression groups of SERPING1 and STEAP3. (F, G) Scatter plots showing the correlations of SERPING1 expression with infiltrated B cells naive (F), macrophage M0 (G) in HCC samples. (H) Overlap showing the results of CIBERSORT algorithms and Spearman algorithms. *p < 0.05; **p < 0.01; ***p < 0.001; green and blue: low‐expression groups; red: low‐expression groups.

In order to investigate the connection between immune cell infiltration and SERPING1 and STEAP3 expression in HCC, we first evaluated the quantity and composition of 22 distinct immune cell types in HCC samples using the CIBERSORT method, as shown in Figure 9A. To further understand the association between genes and invading cells, we examined the differences between the high‐expression and low‐expression groups for 22 immune cell types in the TCGA datasets. A higher proportion of monocytes and a lower proportion of M0 macrophages were associated with increased SERPING1 expression (Figure 9B). Nonetheless, we see no substantial variations in STEP3 expression or immune cell location (Figure 9C). The research found that the high‐expression group of SERPING1 had low levels of expression of a large number of immune checkpoint genes, such as ICOS, TNFRSF4, TNFRSF14, TNFSF4, LGALS9, TNFSF9, CD70, PDCD1, LAG3, CTLA4, TNFRSF18, CD80, CD276, TNFSF15, and NRP1 (Figure 9D). Compared to the group with low expression, the high expression group of STEAP3 exhibited significantly lower expression levels of TNFRSF4, TNFSF4, TNFSF9, TNFSF14, CTLA4, TNFRSF18, TNFRSF9, CD274, and IDO2 (Figure 9E). We further investigated the correlation between immune cells and gene expression by Spearman analysis. The integration of immune cell infiltration data and immune checkpoint variations indicated that intersectional genes, particularly SERPING1, were significantly associated with the progression of the TME. The findings indicate that SERPING1 exhibited a correlation with M0 macrophages (*r* = −0.32, *p* = 0.044) and naïve B cells (*r* = 0.33, *p* = 0.038) (Figure 9F,G). The integration of CIBERSORT and Spearman algorithm findings indicates a significant correlation between cross‐immune cell M0 macrophages and SERPING1 (Figure 9H). These findings indicate the need for further research on these immune cell subgroups' functions and importance in the prognosis and progression of HCC.

### 3.9 Comparison of Drug Sensitivity Between High and Low Expression Levels

**FIGURE 10:**
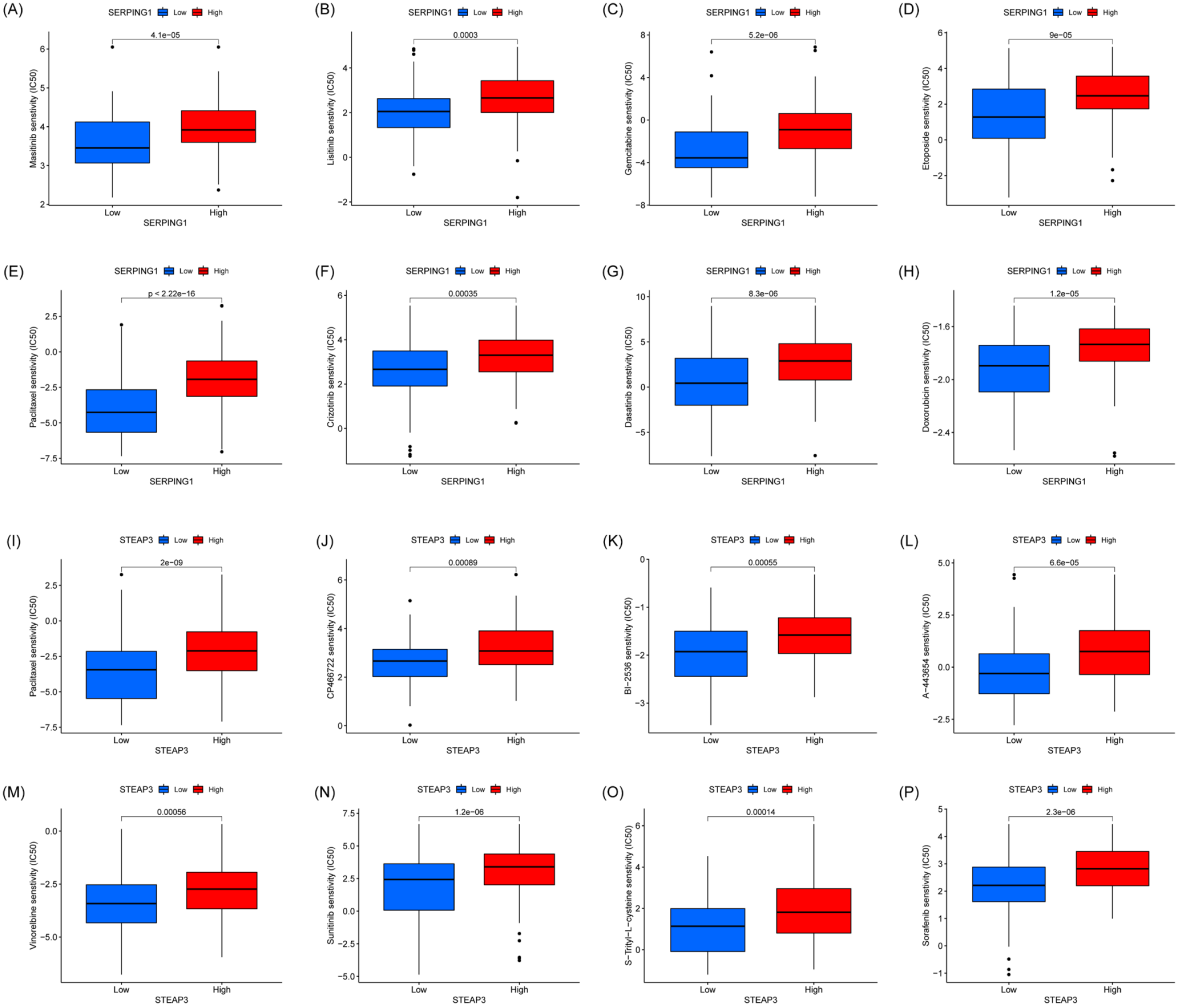
Candidate potential sensitive drugs for low‐ and high‐expression HCC patients. The *y*‐axis represents the IC50 value, which is negatively correlated with drug sensitivity. (A–H) Prediction of chemotherapy drugs for patients grouped according to SERPING1 expression. (I–P) Prediction of chemotherapy drugs for patients grouped according to STEAP3 expression.

We assessed the sensitivity of HCC patients to chemotherapy medications using the “pRRophetic” software program. Individuals in the SERPING1 low expression cohort had heightened sensitivity to Masitinib, Lixitinib, Gemcitabine, Etoposide, Doxorubicin, Dasatinib, Crizotinib, and Paclitaxel (Figure 10A–H). For those in the low expression category, these drugs could improve their prognosis. Patients in the STEAP3 low expression group had increased sensitivity to Paclitaxel, CP466722, BI‐2536, A‐443654, Vinorelbine, Sunitinib, S‐trityl‐L‐cysteine, and Sorafenib, as seen in Figure 10I–P. Administration of these medications may be advantageous for those in the low expression cohort.

### 3.10 Expression of SERPING1 in Liver Cancer Tissues and Cell Lines

**FIGURE 11:**
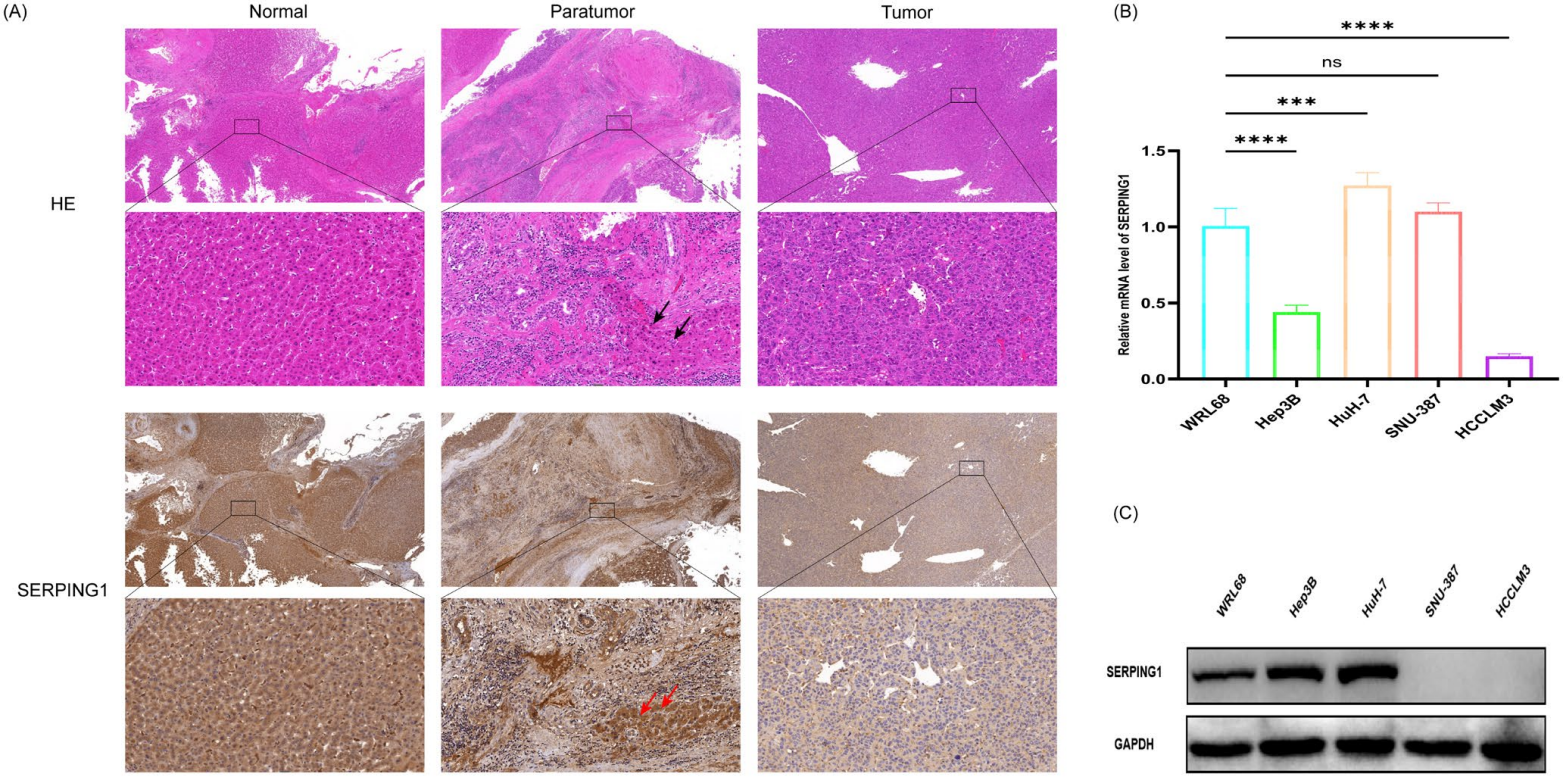
Expression level of SERPING1 in HCC tissues and HCC cell lines. (A) HE and immunohistochemical staining results of SERPING1 in normal, paratumour and cancerous tissues, the arrows show colocalization of normal tissues. (B, C) Protein and mRNA expression levels of SERPING1 were evaluated in HCC cell lines as well as in WRL68 cells. Relative quantification was performed using the 2^−ΔΔCt^ method, with normalisation against GAPDH. ***p < 0.001, ****p < 0.0001.

We assessed the expression level of SERPING1 protein in HCC patients using HE and IHC labelling of HCC tissues, adjacent non‐cancerous tissues, and normal liver tissues. Figure 11A illustrates that HE staining revealed a marked histological distinction between HCC and normal liver tissue. Immunohistochemical study of SERPING1 revealed a significant reduction in its expression in HCC. The arrows indicate that SERPING1 expression in the adjacent normal liver tissue around HCC was markedly elevated compared to that in the cancerous tissue, highlighting the invasive traits of HCC. SERPING1 expression levels in normal liver cell lines and several HCC cell lines were then assessed using Western blot analysis and RT‐qPCR. The mRNA expression levels of Hep‐3B and HCCLM3 were low (Figure 11B), but SERPING1 protein expression was undetectable in SNU‐387 and HCCLM3 (Figure 11C). The findings indicate that SERPING1 may serve an inhibitory function in HCC development and might potentially be used as a novel marker for HCC.

### 3.11 SERPING1 Promotes Proliferation and Invasion of HCC Cells

**FIGURE 12:**
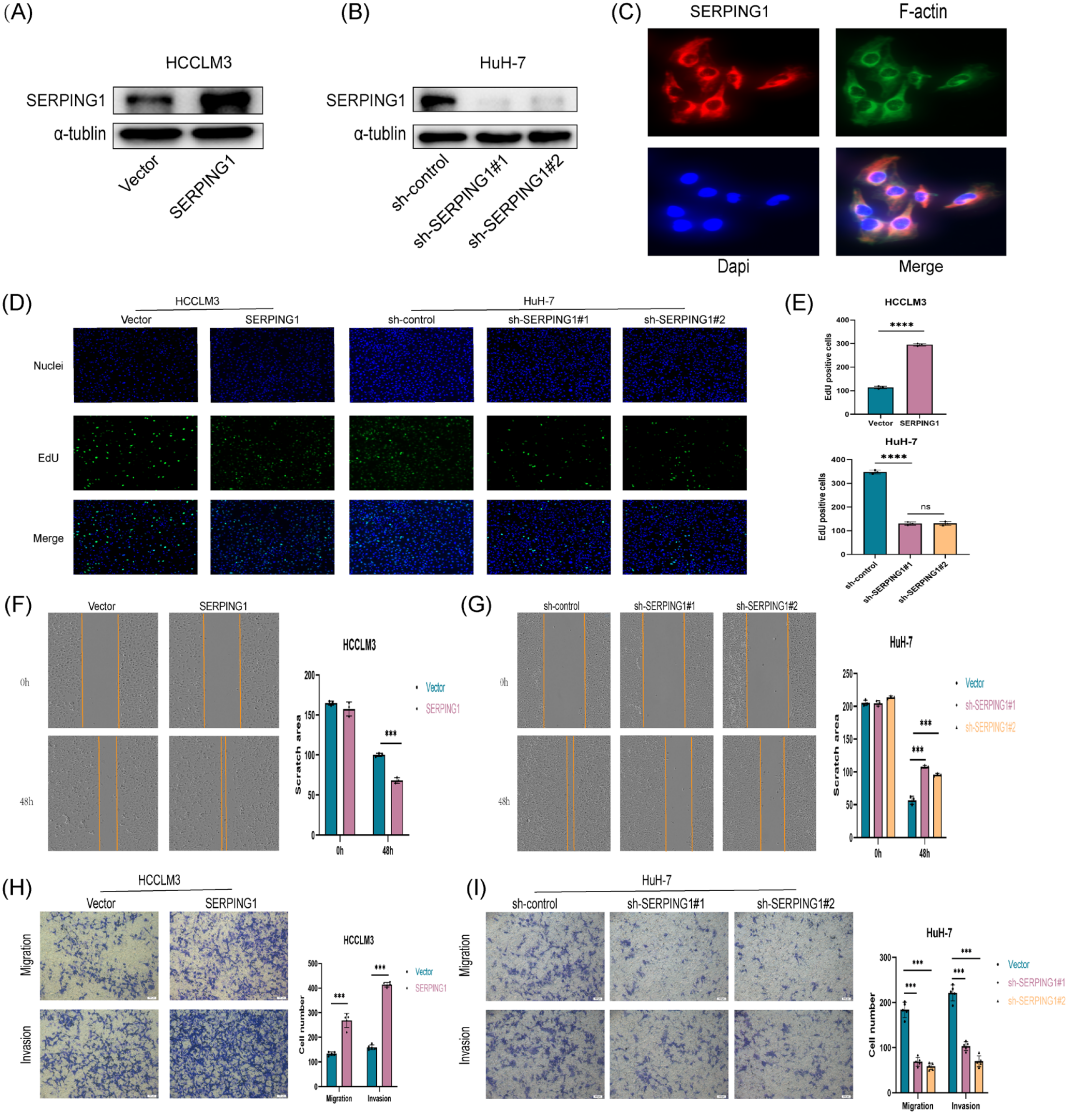
SERPING1 promotes proliferation and invasion of HCC cells. (A, B) Western blot was performed to verify the overexpression and knockdown efficiency of SERPING1. (C) Immunofluorescence showing SERPING1 localization in the cytoplasm of HuH‐7 cells. (D, E) The effect of SERPING1 on the proliferation of HCCLM3 and HuH‐7 cells was detected in EdU assays. (F, G) The migration ability of HCC cells was determined by wound‐healing assays. (H, I) The invasion ability of lung cancer cells was shown by transwell assays. Data are shown as the mean ± SEM of at least three independent experiments. ***p < 0.001, ****p < 0.0001.

Based on the Western blot results, SERPING1 expression was relatively low in HCCLM3 cells and notably higher in HuH‐7 cells. In order to clarify SERPING1's possible function in HCC cells, we initially overexpressed SERPING1 in HCCLM3 cells and silenced it in HuH‐7 cells. The effectiveness of SERPING1 knockdown and overexpression was then verified by Western blot analysis, which demonstrated significant alterations in SERPING1 expression following these treatments (Figure 12A,B). Additionally, immunofluorescence staining revealed that SERPING1 is predominantly localised in the cytoplasm (Figure 12C). EdU assays further showed that SERPING1 overexpression promoted cell proliferation, while its silencing inhibited proliferation (Figure 12D,E). Assays for wound healing, transwell migration, and Matrigel invasion were used to evaluate the effect of SERPING1 on cell migration and invasion. The overexpression of SERPING1 markedly increased the migratory and invasive abilities of HCCLM3 cells (Figure 12F,H), whereas SERPING1 knockdown markedly suppressed these abilities in HuH‐7 cells (Figure 12G,I). All things considered, the results imply that SERPING1 is essential for accelerating the growth of HCC cells in vitro.

## 4 Discussion

As the most prevalent kind of liver cancer, hepatocellular carcinoma is one of the main causes of cancer‐related mortality globally. Notwithstanding advancements in therapy, the prevalence of liver cancer persists in escalating globally. Curative treatments, including hepatectomy or liver transplantation, are viable just for a restricted group of individuals identified at an early stage of HCC. This work aims to find potential genes using a thorough investigation of TCGA and GEO databases, together with integrated Mendelian Randomisation analysis of GWAS and eQTL data derived from peripheral blood. We provide novel insights into the possible targets and molecular processes of HCC using functional enrichment analysis, signalling pathway analysis, and immune cell infiltration study, along with options for therapeutic therapy.

We examined three microarray datasets to discover genes linked to HCC, including 488 samples from the GEO database and 374 samples from TCGA. By integrating this data with eQTL information and results from Jiang et al. [16], which included 456,348 HCC patients, we performed a thorough analysis that identified two genes, SERPING1 and STEAP3, as possible critical contributors to HCC aetiology.

The next study is to investigate the functions of two recently discovered genes, SERPING1 and STEAP3, which may have substantial consequences in the aetiology of HCC. SERPING1, situated on chromosome 11, encodes a 55 kDa plasma protein termed C1‐inhibitor, a highly glycosylated entity that modulates the conventional complement system [22, 23]. Complement activation in cancer has a dual purpose: it may eliminate tumour cells by augmenting antibody‐dependent and complement‐dependent cytotoxicity [24], it may further facilitate tumour advancement within the tumour microenvironment [25, 26, 27]. The role of SERPING1‐mediated classical pathway activation in either facilitating tumour cell eradication or enhancing tumour proliferation in HCC remains ambiguous. The STEAP protein family, which includes STEAP1, STEAP2, STEAP3, and STEAP4, is a group of mammal‐specific transmembrane proteins exhibiting metal reductase activity, highlighting its significance in metal metabolism [28]. STEAP3 is specifically associated with the advancement of many malignancies, such as colorectal [29], ovarian [30], and lung squamous carcinoma [31]. Recent studies have emphasised STEAP3's regulatory role in ferroptosis via iron metabolism [32], establishing it as a pivotal factor in cancer biology. Nonetheless, its significance in HCC prognosis and immunological infiltration remains mostly unexamined [33, 34].

This research undertook a thorough examination of the functions and mechanisms of SERPING1 and STEAP3 in HCC. Our results indicate that both SERPING1 and STEAP3 are repressed in HCC tissues. In order to determine their impact on the development and prognosis of HCC, we investigated the connection between clinical features and expression levels in HCC patients using extensive clinical data from TCGA. We used single‐cell RNA sequencing to examine gene expression at the single‐cell level, allowing for the analysis of transcriptomes in individual cells from HCC samples. This methodology yields vital insights into gene expression at the single‐cell level, crucial for comprehending cellular heterogeneity and gene regulatory mechanisms, hence elucidating their functional functions and regulatory pathways in HCC development [35, 36]. However, we acknowledge that the TME in HCC is highly heterogeneous. Differences in patient populations, tumour stages, and sequencing platforms across datasets could impact the expression patterns of these genes and their roles in the TME. Future studies should validate these findings using additional independent datasets to better understand the generalizability and biological significance of SERPING1 and STEAP3 in HCC. To elucidate the biological roles of SERPING1 and STEAP3 in HCC, we performed GO and KEGG enrichment analysis for each gene. GSEA was also used to identify essential signalling pathways and biological activities. Ultimately, Pearson correlation analysis indicated that several genes co‐expressed with SERPING1 and STEAP3, regardless of their positive or negative association, had substantial prognostic significance for patients with HCC.

We conducted a more in‐depth examination of the functions of SERPING1 and STEAP3 inside the tumour microenvironment of HCC. CIBERSORT analysis showed significant variations in the location of immune cells between groups with SERPING1 expression, but not between groups with high and low STEAP3 expression. We then used Pearson correlation analysis to assess the link between SERPING1 and immune cell populations, revealing a substantial association between SERPING1 and M0 macrophages in both methodologies. This discovery indicates that SERPING1 may regulate the tumour microenvironment by affecting macrophage polarisation. Macrophage polarisation, especially the dynamic shift between M1 and M2 states, is influenced by several stimuli, including cytokines, chemokines, and extracellular matrix components [37]. M1 macrophages are mostly pro‐inflammatory and have anti‐tumour properties by releasing effector molecules including nitric oxide and tumour necrosis factor, which are essential for pathogen elimination, inflammation, and tissue injury [38]. M2 macrophages are often linked to anti‐inflammatory and pro‐tumour activities, facilitating tissue repair, angiogenesis, immunosuppression, and tumour advancement via the release of regulatory substances such as ornithine and interleukin‐10. The equilibrium and functional states of M1 and M2 macrophages vary under many physiological and pathological situations, affecting immune response results [39, 40].

Advancements in immunogenomics have revealed a growing array of immune‐related genes as potential therapeutic targets [41]. We found 15 genes associated with SERPING1 and 9 associated with STEAP3 by the comparison of 40 common immune checkpoint genes' expression levels between cohorts with high and low expression. Correlation study of immune infiltration indicates that specific immunotherapeutic strategies may be customised according on immune‐related gene expression patterns among different groups. The data suggest that SERPING1 and STEAP3 may be new genes with crucial roles in the development of HCC and potential therapeutic targets. Consequently, we examined prospective therapeutic drugs. Our data reveals that patients with elevated SERPING1 expression have heightened sensitivity to Lisitinib, gemcitabine, and analogous substances, while those with high STEAP3 expression showed enhanced sensitivity to vinorelbine, sorafenib, and comparable medications. Our study identified potential chemotherapy agents for HCC patients based on differential sensitivity between high‐ and low‐expression cohorts of SERPING1 and STEAP3. The identified drugs, including Paclitaxel, Dasatinib, Sorafenib, and others, are well‐established in the context of cancer treatment. While compounds such as benzopyran, benzamidocoumarin, and barbituric acid derivatives have been explored as anti‐tubercular agents, their relevance to HCC treatment remains unclear and was not within the scope of our analysis. In addition to the identification of SERPING1 and STEAP3 as potential therapeutic targets in HCC, it is crucial to explore existing drugs or compounds that could be repurposed to target these genes. Given the potential roles of SERPING1 in complement system regulation and STEAP3 in iron metabolism, there are existing therapeutic avenues that could be explored to target these proteins in HCC. SERPING1 has long been studied for its role in regulating the complement system, with C1‐inhibitors already used in complement‐related diseases, such as paroxysmal nocturnal hemoglobinuria, and potentially applicable in cancer immunotherapy [42]. Additionally, targeting the iron metabolism pathway, through iron chelators, has shown promise in cancer therapies, especially in prostate and liver cancers, as STEAP3 is crucial for iron homeostasis in tumour cells [43]. These approaches, including the repurposing of FDA‐approved drugs such as deferoxamine, could offer novel strategies for targeting SERPING1 and STEAP3 in HCC. Due to the little research on SERPING1 in HCC, we also validated its expression at both the transcriptional and protein levels in hepatocellular carcinoma tissues and cell lines, and we preliminarily verified the cancer‐promoting role of SERPING1 in HCC cells.

Although our research produces significant results, many critical limitations must be recognised: (1) Our study relies on retrospective datasets, which introduce potential biases. Selection bias may occur as patient cohorts in these datasets are not always representative of the broader population. Additionally, while we adjusted for known confounders, residual confounding from unmeasured variables (e.g., lifestyle factors) remains a concern. Furthermore, the lack of temporal information limits our ability to make strong causal inferences. Prospective studies with more detailed longitudinal data are needed to confirm our findings and improve their generalizability. (2) Although MR is a robust method for causal inference, it has inherent limitations. Horizontal pleiotropy (where genetic variants affect outcomes through pathways unrelated to the exposure) may bias results, despite sensitivity analyses like MR‐Egger. Additionally, weak instrumental variables could lead to imprecise or biased estimates.

## 5 Conclusions

Using the most recent GWAS and eQTL data, bulk RNA sequencing, and single‐cell RNA sequencing, we identified important genes and pathways connected to SERPING1 and STEAP3 that are related to HCC. We used modern bioinformatics and statistical techniques to analyse their possible activities and clinical significance, highlighting their crucial involvement in the immunological microenvironment. This research elucidates the intricate molecular pathways and prospective therapies for HCC.

## Author Contributions

**Mingkai Gong:** writing – original draft (equal). **Xian Zhao:** writing – original draft (equal). **Qingze Li:** writing – original draft (equal). **Qisheng Hao:** data curation (equal), formal analysis (equal), software (equal). **Lichao Cha:** formal analysis (equal), software (equal). **Guofei Dong:** formal analysis (equal), software (equal). **Xinyu Li:** formal analysis (equal), software (equal). **Fabo Qiu:** formal analysis (equal), software (equal). **Dan Li:** supervision (equal), validation (equal), writing – review and editing (equal). **Lantian Tian:** supervision (equal), validation (equal), writing – review and editing (equal).

## Ethics Statement

This study was approved by the ethics committee of the Affiliated Hospital of Qingdao University and carried out under the World Medical Association Declaration of Helsinki.

## Consent

Informed consent was obtained from all subjects involved in the study.

## Conflicts of Interest

The authors declare no conflicts of interest.

## Supporting information


Figure S1

## Data Availability

The authors are able to provide the data generated by the analysis of this study upon reasonable request.
