# Supplementary material for: Integrated Analysis of Bulk RNA Sequencing, eQTL, GWAS, and Single‐Cell RNA Sequencing Reveals Key Genes in Hepatocellular Carcinoma
Source: J Cell Mol Med. 2025 Jan 23;29(2):e70359. doi: 10.1111/jcmm.70359 (PMC11756993; doi:10.1111/jcmm.70359)
Supplement: Supplementary file 1 — Figure S1 [file JCMM-29-e70359-s001.docx]

**
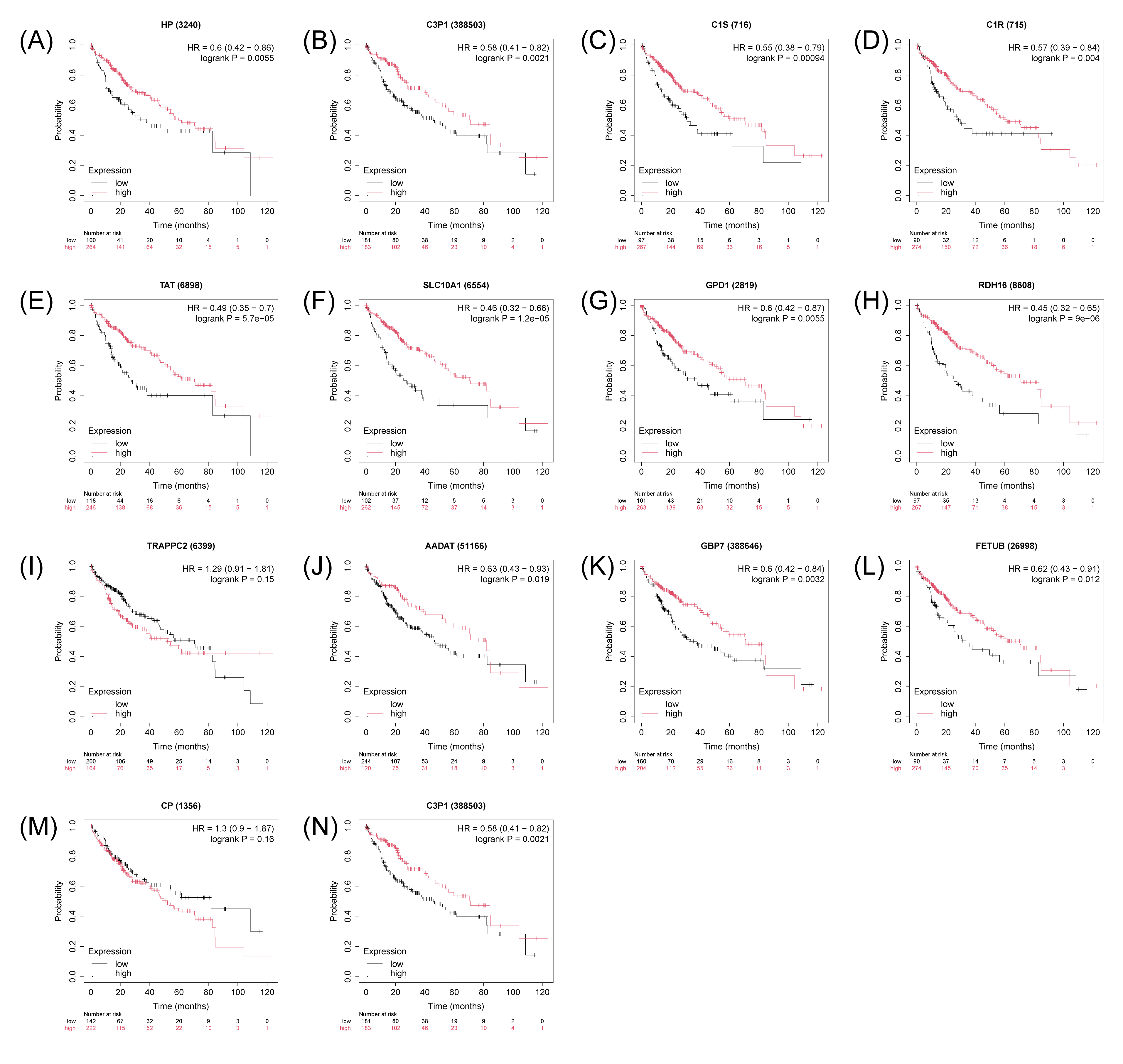
**

**SUPPLEMENT FIGURE** Kaplan–Meier curve of co-expressed genes.

1. H) co-expressed genes of SERPING1.

(B-N) co-expressed genes of STEAP3.
